# Dysregulated signaling, proliferation and apoptosis impact on the pathogenesis of TCRγδ+ T cell large granular lymphocyte leukemia

**DOI:** 10.1371/journal.pone.0175670

**Published:** 2017-04-13

**Authors:** Martine J. Kallemeijn, Dick de Ridder, Joyce Schilperoord-Vermeulen, Michèle Y. van der Klift, Yorick Sandberg, Jacques J. M. van Dongen, Anton W. Langerak

**Affiliations:** 1 Laboratory of Medical Immunology, Department of Immunology, Erasmus Medical Center Rotterdam, Rotterdam, The Netherlands; 2 Department of Bioinformatics, Wageningen University, Wageningen, The Netherlands; Kyung Hee University, REPUBLIC OF KOREA

## Abstract

TCRγδ+ T-LGL leukemia is a rare form of chronic mature T cell disorders in elderly, which is generally characterized by a persistently enlarged CD3+CD57+TCRγδ+ large granular lymphocyte population in the peripheral blood with a monoclonal phenotype. Clinically, the disease is heterogeneous, most patients being largely asymptomatic, although neutropenia, fatigue and B symptoms and underlying diseases such as autoimmune diseases or malignancies are also often observed. The etiology of TCRγδ+ T-LGL proliferations is largely unknown. Here, we aimed to investigate underlying molecular mechanisms of these rare proliferations by performing gene expression profiling of TCRγδ+ T-LGL versus normal TCRγδ+ T cell subsets. From our initial microarray dataset we observed that TCRγδ+ T-LGL leukemia forms a separate group when compared with different healthy control TCRγδ+ T cell subsets, correlating best with the healthy TemRA subset. The lowest correlation was seen with the naive subset. Based on specific comparison between healthy control cells and TCRγδ+ T-LGL leukemia cells we observed up-regulation of survival, proliferation and hematopoietic system related genes, with a remarkable down-regulation of apoptotic pathway genes. RQ-PCR validation of important genes representative for the dataset, including apoptosis (*XIAP*, *CASP1*, *BCLAF1* and *CFLAR*), proliferation/development (*ID3*) and inflammation (*CD28*, *CCR7*, *CX3CR1* and *IFNG*) processes largely confirmed the dysregulation in proliferation and apoptosis. Based on these expression data we conclude that TCRγδ+ T-LGL leukemia is likely the result of an underlying aberrant molecular mechanisms leading to increased proliferation and reduced apoptosis.

## Introduction

T cell large granular lymphocytic (T-LGL) leukemia is a heterogeneous chronic mature T cell neoplasia, which is recognized as a separate hematological disorder according to the World Health Organization (WHO) classification [1]. T-LGL leukemia originates from normal LGL cells which comprise 10–15% of peripheral blood mononuclear cells (PBMCs) [2] and can be subdivided into two major groups based on the type of T cell receptor (TCR): TCRαβ or TCRγδ. The majority of T-LGL leukemia involves the TCRαβ+ CD8+ variant (80–90%), while only a small part of the T-LGL leukemia has a TCRαβ+CD4+ (1–5%) or TCRγδ+ phenotype (5%) [3,4]. TCRγδ+ T-LGL leukemia is a chronic and heterogeneous disorder, which in fact comprises a spectrum–from lymphoproliferative disease to leukemia–and generally shows an indolent disease course, affecting mostly elderly patients with an average age of 60 years [5–7]. Approximately one-third of the patients is asymptomatic at diagnosis [8,9]. Most clinical features concern neutropenia, recurrent bacterial infections and B symptoms that are associated with chronic leukemia. Typically, TCRγδ+ T-LGL leukemia is highly associated with cytopenia, autoimmune diseases such as rheumatoid arthritis, and malignancies varying from other hematological cancers to solid tumors [4]. The diagnosis is based on a persistent (>6 months) monoclonal CD3+/CD57+/TCRγδ+ LGL population (>0.4 x 10^9^/L) in the peripheral blood (PB) and/or bone marrow (BM), confirmed by flow cytometry or cell morphology [9]. Furthermore, TCRγδ+ T-LGL leukemia is mostly CD4-negative, partly CD8-positive, and approximately 50% show CD16 and CD56 expression [6]. Despite recent advances for T-LGL leukemia in general, the disease etiology of TCRγδ+ T-LGL leukemia remains largely unknown. It has been hypothesized that chronic (antigenic) stimulation would play a major role in the development of the proliferation, as has actually been shown for the TCRαβ+CD4+ T-LGL leukemia type [10]. Also, in 2006 Sandberg *et al*. identified the presence of the so-called invariant T selection determinant leading to a conserved amino acid at the relative second position of the CDR3 region in Vδ2 –Jδ1 rearrangements in a large subgroup of TCRγδ+ T-LGL proliferations, indicating that these cells are antigen-experienced. Furthermore, an activation-associated effector phenotype and a skewed and dynamic TCR repertoire–also referred to as clonal drift [11]–have been found in T-LGL proliferations, again suggestive of a role for antigens, but the exact antigen or the type of antigens have so far not been elucidated [3,12]. Even though the involved antigen(s) are unknown and might be variable, and the disease is clinically heterogeneous, we hypothesized that TCRγδ+ T-LGL leukemia patients have common underlying molecular defects that contribute to the leukemogenesis. In order to obtain more in-depth insights into the mechanisms driving these TCRγδ+ T-LGL proliferations we therefore performed gene expression profiling analysis in purified TCRγδ+ T-LGL leukemia samples. Here we present data that TCRγδ+ T-LGL leukemia cells originate from the most common antigen-experienced TCRγδ+ T cell population in the adult peripheral blood, and that they have undergone a transformation leading to an imbalance in proliferation and apoptosis, eventually contributing to the TCRγδ+ T-LGL leukemia pathogenesis.

## Materials and methods

### Patients and healthy controls

The database files from the department of Immunology, Erasmus MC, University Medical Center (Rotterdam, The Netherlands) were retrospectively reviewed for cases with a proven mature persistent (>6 months) TCRγδ+ T cell proliferation in PB and/or BM based on a combination of clinical, histological (HE sections), cytomorphological (May-Grünwald-Giemsa staining), laboratory, immunophenotypical (including for the majority of cases most of the following markers: CD3, TCRγδ, CD4, CD8, CD16, CD56, CD57, CD45RA, CD45RO, CD27, CD197) and molecular data (monoclonal TRG and TRD gene rearrangements) [13,14]. Other TCRγδ+ T cell proliferation diseases such as hepatosplenic lymphomas were excluded based on clinical, cytomorphological and/or histopathological data. From 10 TCRγδ+ T-LGL leukemia cases frozen cell material was available for inclusion in the current study. The immunophenotypical and molecular analyses were performed on PB and/or BM samples which were erythrocyte-lysed using FACS lysing solution (BD Biosciences, San Jose, CA, USA). PB- or BMMC were isolated by means of Ficoll-Paque (density 1.077 g/ml, Pharmacia, Uppsala, Sweden). As controls, healthy blood donors from Sanquin Blood Bank (Amsterdam, The Netherlands) were included upon informed consent, which were anonymized for further use. PBMCs were obtained with Ficoll density gradient separation and cryopreserved in Iscove’s Modified Dulbecco’s Medium (IMDM, Lonza, Basel, Switzerland) with dimethyl sulfoxide and stored in vials at -180°C until further use.

Use of all samples for the study was approved by the Erasmus MC Medical Ethics Committee (MEC-2015-617). Studies were conducted in accordance with the principles of the Declaration of Helsinki.

### Immunophenotyping and cell sorting

Control PB samples were immunophenotyped to examine the composition of TCRγδ+ T cell subsets based on membrane marker expression, including CD3, CD4, CD8, CD19, CD27, CD45RA, CD45RO, CD27, CD197, TCRαβ, TCRVδ1 and TCRVδ2 (S1 Table). Measurements were performed with a FACS Canto II or FACS LSR Fortessa flow cytometer (BD Biosciences). The immunophenotype of the LGL proliferations was already determined during diagnostic work-up, and was therefore available in the database.

For sorting experiments cryopreserved PB- and/or BMMCs were thawed and sorted. Sorting was performed specifically on the tumor cells from the patient samples, using CD3, CD45, TCRαβ, TCRγδ, TCRVδ1 and TCRVδ2 markers.

Healthy control samples were sorted into five different CD3+TCRγδ+ T cell subsets based on gating strategies in S1 Fig: total TCRVδ1, total TCRVδ2, effector memory (= TemRO population, CD45RO+CD27-CD197-), effector (= TemRA population, CD45RA+CD27-CD197-) subsets and a naive, non-antigen stimulated control (CD45RA+CD27+CD197+) subset. Sorting was performed with a FACS Aria I or III (BD Biosciences).

### RNA isolation, cDNA synthesis

After sorting the cells were lysed and subjected to combined DNA/ RNA isolation with the QIAGEN DNA/RNA/miRNA AllPrepKit (QIAGEN, Hilden, Germany).

For the gene expression profiling experiments RNA from the sorted healthy control TCRγδ+ T cell subsets was pooled in order to obtain higher amounts of RNA and to create pooled healthy control subset samples; N = 3 for Vδ1 and Vδ2 subsets, N = 8 for naive, TemRO and TemRA subsets due to lower sorting yields.

For RQ-PCR tests cDNA was synthesized from isolated RNA as well as cryopreserved RNA with reverse transcriptase Superscript II (Invitrogen Life Technologies, Waltham, MA, USA), 10x CA buffer (0.2 M Tris pH 8.3, 0.5M KCl), dNTP (GE Healthcare, Cleveland, OH, USA), dithiotreitol (Invitrogen Life Technologies), MgCl_2_ (Applied Biosystems Life Technologies, Waltham, MA, USA), recombinant RNAsin (Promega, Fitchburg, WI, USA) and random primers (Invitrogen Life Technologies).

### Gene expression profiling

Isolated RNA from sorted patient tumor cells and the 5 control TCRγδ+ T cell subsets was further amplified, reverse transcribed into cDNA, purified, fragmented, biotinylated and hybridized to Affymetrix HG-U133 Plus 2.0 GeneChip arrays (containing 54,675 probe sets) according to the Affymetrix GeneChip 3’ IVT Express Kit user manual (Affymetrix, Santa Clara, CA, USA), as described previously [15–17]. Robust multi-array average (RMA) background removal, compensation for systematic technical differences, quantile normalization and probe set summary were performed [18]. Unsupervised correlation and clustering plots were made based on selected probe sets that showed signal, i.e. for which the median absolute deviation (MAD) from the median exceeded a certain threshold *Thr* on a log_2_ scale. To further assess differential expression, a number of supervised analyses were applied: fold change calculation (FC), analysis of variance (ANOVA) [19] and significance analysis of microarrays (SAM) were applied [20]. All comparisons concerned disease cases versus normal control cases. Data were analyzed through the use of the Database for Annotation, Visualization, and Integrated Discovery (DAVID Database) [21,22], and QIAGEN’s Ingenuity Pathway Analysis (IPA, QIAGEN, Redwood City, USA; www.qiagen.com/ingenuity).

### Real-time Quantitative PCR (RQ-PCR)

Assays were designed with the Roche Universal Probe Library (Roche, Basel, Switzerland) (S2 Table). RQ-PCR experiments were performed with TaqMan Universal PCR master mix (2x) (Applied Biosystems) on the StepOnePlus instrument (Thermo Fisher, Waltham, MA, USA). Ct values of disease samples were normalized to the Ct value of the ABL housekeeping gene [23] and normalized Ct values of healthy control samples (ΔΔCt method).

### Statistical analysis

Supervised statistical analyses for the gene expression profiling data were performed using analysis of variance (ANOVA), with significance cutoff at p<0.05 and as threshold of up- and/or down-regulation ≥2 fold change. Significance of microarray analysis (SAM) with significance cutoff <0.05 was included in the analysis as comparison. Bonferroni and Benjamini-Hochberg p-value adjustment were applied for multiple testing correction and comparison of different statistical tests on the data set.

## Results

### Heterogeneity in clinical presentation, associated diseases and immunophenotype and–genotype among TCRγδ+ T-LGL leukemia patients

In all ten patients a proven TCRγδ+ T-LGL leukemia was identified, based on immunophenotype, immunogenotype, high leukocyte count, and high percentage of TCRγδ+ T-LGL cells compared with reference values of PB TCRγδ+ T cells (<5% of all lymphocytes in general) [6]. All cases were diagnosed as TCRγδ+ T-LGL leukemia, while other causes of TCRγδ+ T-cell proliferation, such as hepatosplenic lymphoma, were excluded. There was a slight male predominance among the patients (seven males, three females). Most patients showed chronic leukemia-associated symptoms, such as fever and cytopenia, and underlying (auto)immune diseases such as rheumatoid arthritis, Graves’ disease and uveitis (Table 1). In general, immunophenotyping showed CD3-positivity, CD4-negativity and in some cases CD8-positivity, together with variable expression of markers that have been associated with LGL cells such as CD16, CD56 and/or CD57, and frequent CD45RA positivity in combination with CD27 and/or CD197 negativity, pointing towards TemRA and TemRO phenotypes (Table 1). For some patients Vγ and Vδ-usage was determined with flow cytometry as well, which correlated with the immunogenotyping data (Table 1). Among the patients half showed Vδ1 –Jδ1 rearrangements, whereas the other half showed Vδ2 –Jδ1 rearrangements. Most patients had a Vγ9 –Jγ1.1/2.1 or Jγ1.3/2.3 rearrangement, leading to an overall receptor predominance of Vγ9/Vδ1 and Vγ9/Vδ2. Most patients showed a monoclonal TCRγδ+ T-LGL cell population at the receptor level, whereas two patients showed a somewhat oligoclonal profile albeit with a dominant clone, possibly indicating the presence of additional non-aberrant TCRγδ+ T cells or subclones (Table 1).

**Table 1:** TCRγδ+ T-LGL leukemia patient characteristics and immunogenotypic features.

	Phenotype						Genotype						
							TRD rearrangement‡			TRG rearrangement‡			
Patient	Gender	Age at diagnosis (years)	Clinical presentation and associated disease	Immunophenotype	Tumor load (%leukocytes)	Absolute LGL count (10^9^/ml)	TRDV	TRDD	TRDJ	TRGV	TRGJ^+^	Overall receptor	Clonality^x^
LGL056	F	40	Anemia, neutropenia, M. Graves	CD3+/CD4-/CD8+/CD16+/ CD56+/ CD57+/ CD45RA+/CD45RO-/Vγ9+/Vδ1+	41	3.6	1*01	2*01/ 3*01	1*01	9*01	1*02	Vγ9/Vδ1	Monoclonal
LGL057	M	46	Fever, positive Mantoux test	CD3+/CD4+/CD8+/CD56+/Vγ9+/Vδ2+	22.7	0.8–1.2	2*01/ 02/03	3*01	1*01	9*01	P1*01	Vγ9/Vδ2	Monoclonal
LGL058	M	38	Lymphadenopathy, uveitis, sarcoidosis	CD3+/CD4/CD16+/CD56+/CD57+/CD45RA+/CD27-/Vδ2+	6.9	3.4	2*01/ 02/03	3*01	1*01	9*01	1*02	Vγ9/Vδ2	Monoclonal
LGL063	M	56	Unknown	CD3+/CD4- /CD8+/CD16+/CD56+/CD57+/CD27-/CD197-/Vδ1+	23	1.4	1*01	2*01/ 3*01	1*01	9*01	P1*01	Vγ9/Vδ1	Oligoclonal
LGL064	M	76	Anemia, thrombocytopenia, hepatosplenomegaly, rheumatoid arthritis	CD3+/CD4+/CD8+/CD16+/CD56+/CD57-/CD45RA+/CD45RO+/CD27+/Vδ1+/Vδ2-	24	1.7	1*01	2*01/ 3*01	1*01	4*01	1*01/02/ 2*01	Vγ4/Vδ1	Monoclonal
LGL083	F	30	Unknown	CD3+/CD4-/CD8+/CD16+/CD57+/CD45RA+/CD27-/CD197-/Vγ9-/Vδ2+	8	0.3–2.4	2*01/ 02/03	3*01	1*01	8*01	2*01	Vγ8/Vδ2	Oligoclonal
LGL087	F	74	Unknown	CD3+/CD4-/CD8+/CD56+/Vδ1+	28	n.d.	1*01	2*01/ 3*01	1*01	2*02	1*01	Vγ2/Vδ1	Monoclonal
LGL088	M	54	Unknown	CD3+/CD4-/CD8-/CD16-/CD56+/CD57+/CD45RA+/CD27-/CD197-/Vδ1+	26	0.8	1*01	3*01	1*01	2*01	P2*01	Vγ2/Vδ1	Monoclonal
LGL089	M	54	Unknown	CD3+/CD4-/CD8+/CD16+/CD56+/CD57+/CD45RA+/CD27-/CD197-/Vγ9+/Vδ2+	10	4.3	2*02	2*01/ 3*01	1*01	9*01	P1*01	Vγ9/Vδ2	Monoclonal
LGL113	M	70	Unknown	CD3+/CD4-/CD8-/CD16-/CD56-/CD57-/CD45RA+/CD45RO+/CD27-/CD197-/Vγ9+/Vδ2+	51	6	2*01/ 02/03	2*01/ 3*01	1*01	9*01	1*01/02/ 2*01	Vγ9/Vδ2	Monoclonal

^‡^ Productive and in-frame rearrangements are shown. N.d., not determined.

^+^TRGJ annotations according to the IMGT nomenclature [24]. TRGJP1: Jγ1.1, TRGJP2: Jγ2.1, TRGJ1: Jγ1.3, TRGJ2: Jγ2.3. The canonical TRGJP (Jγ1.2) was not included in the diagnostic work-up with IVS TRG multiplex PCR (Invivoscribe).

^x^Based on GeneScan and heteroduplex analysis after multiplex PCR.

### Unsupervised clustering of TCRγδ+ T-LGL leukemia cases shows highest resemblance to normal effector TCRγδ+ T cells

**Fig 1:**
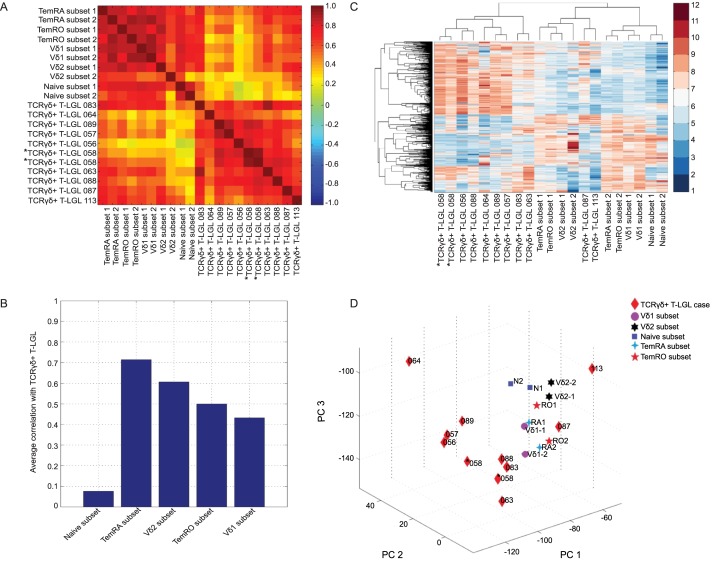
Unsupervised gene expression data analysis. (A) Heatmap analysis shows clear correlations between TCRγδ+ T-LGL proliferation cases, and lower correlations to the healthy control TCRγδ+ T cell subsets, (B) the highest being to the TemRA TCRγδ+ T cell subset. (C) The clustergram shows that the TCRγδ+ T-LGL proliferation cases indeed form a distinct group, except for two cases which cluster closer to healthy control TemRA, TemRO and TCRVδ2 TCRγδ+ T cell subsets. (D) Principal component analysis (PCA) in three-dimensional (3D) graph shows similar clustering results. Thr = 0.7; 1336 probe sets. Case LGL058 indicated in italics was studied in duplo to assess reproducibility of the assay.

Despite some level of phenotypical heterogeneity between patients, we next evaluated the transcriptomes of ten TCRγδ+ T-LGL leukemia patients versus five healthy control TCRγδ+ T cell subsets in order to obtain more insight into the immunobiology of the disease and to possibly identify common downstream pathogenic events in TCRγδ+ T-LGL leukemia. As from literature it is known that TCRγδ+ T-LGL leukemias are antigen-experienced [4,6], TemRO and TemRA TCRγδ+ subsets were used as healthy control counterparts for the TCRγδ+ T-LGL cells. As a non-antigen stimulated control the naive TCRγδ+ population was included. In order to get a general view from gene expression profiling, first a correlation analysis was performed based on selected probe sets that showed significant variation between all microarrays (with a median absolute deviation from the median (MAD) of at least *Thr* = 0.7). In the heatmap TCRγδ+ T-LGL leukemia cases and healthy control TCRγδ+ T cell subsets were on purpose represented separately, as they represent distinct groups. The heatmap showed a clear distinction between TCRγδ+ T-LGL leukemia cases and healthy control TCRγδ+ T cell subsets, suggesting that the TCRγδ+ T-LGL leukemia cases indeed form a separate group with a distinct transcriptome (Fig 1A). The healthy control subset that correlated best with the TCRγδ+ T-LGL leukemia cases was the TemRA TCRγδ+ T cell subset, and to a lesser extent the TemRO subset, in line with our patient phenotype data (Table 1), whereas the naive TCRγδ+ T cell subsets showed the lowest correlation, as was expected (Fig 1B). Hierarchical clustering confirmed that 8/10 TCRγδ+ T-LGL leukemia cases indeed formed a separate group from the healthy control subsets, whilst two cases, LGL087 and LGL113, grouped together with TemRA, TemRO and TCRVδ2 subsets (Fig 1C). The same clustering was observed when a principal component analysis (PCA) was performed (Fig 1D). One case was studied in duplo (LGL058, Fig 1) to assess reproducibility of the arrays, which was indeed high. Overall the TCRγδ+ T-LGL leukemia cases thus mostly form a separate group based on their gene expression profile, showing closest correlation with the healthy normal TemRA TCRγδ+ T cell subset.

### Supervised analysis showed a dysregulated balance in proliferation and apoptosis

Following unsupervised clustering analysis, specific comparisons were performed between all TCRγδ+ T-LGL leukemia cases vs. naive, TemRO and TemRA healthy control TCRγδ+ T cell subsets. Two statistical comparisons, significance analysis of microarrays (SAM) and two-way analysis of variance (ANOVA) were applied that yielded varying numbers of differentially expressed probe sets (S3 Table). Stringent statistical filtering using SAM showed low numbers of mainly down-regulated genes in the comparison between TCRγδ+ T-LGL leukemia cases and TemRO and TemRA subsets. When the comparison was performed with the naive subset, the numbers of differentially expressed probe sets found by both statistical analysis methods were high. Both methods confirmed the relatively high similarity of TCRγδ+ T-LGL leukemia cases to the healthy TemRO and TemRA subsets, and the lower similarity to naive TCRγδ+ T cells as also observed in the unsupervised clustering. Since the SAM analysis showed low numbers of differentially expressed probe sets, and in order to reduce the risk of missing data, we therefore used the two-way ANOVA set for further supervised analysis. Top up- and down-regulated genes in TCRγδ+ T-LGL leukemia cells vs. the best correlating subset, the TemRA TCRγδ+ T cells, are depicted in S4 Table. Most up-regulated genes appeared to be involved in the inflammatory response and response to bacteria: *LYZ*, *S100A8*, *S100A9*, *ALOX5* and *LTB* according to the DAVID database. The top down-regulated genes were mostly associated with the process of transcription; these genes included amongst others *GTF2H3*, *CTBP2* and *ZNF260*.

In order to identify genes associated with biologically more relevant pathways and processes, the probe sets that were significantly differentially expressed with a fold change of at least 2 (both up- and down-regulated) in TCRγδ+ T-LGL leukemia cells when compared to the healthy TemRA TCRγδ+ T cell subset were then further analyzed with the DAVID database [21,22]. Gene Ontology biological process analysis showed processes involved in the regulation of the immune system and response, T cell activation, and response to stress to be affected (Table 2). Enrichment analysis of KEGG pathways in DAVID showed that differentially expressed genes were largely involved in the hematopoietic cell lineage, osteoclast differentiation, rheumatoid arthritis disease and NFκB signaling, although after correction for multiple testing none of these were found to be significant. Nevertheless, similar processes, such as hematopoietic system and inflammation, were identified (Table 3). Differentially expressed probe sets in the comparison between TCRγδ+ T-LGL leukemia cells vs. TemRO cells showed similar processes to be affected (S5 Table). Taken together, both annotation enrichment analyses (Gene Ontology, KEGG) indicated that proliferation, cell survival signaling and inflammatory processes were affected.

**Table 2:** Gene ontology biological process annotation analysis of TCRγδ+ T-LGL leukemia cases versus healthy TCRγδ+ TemRA cells in DAVID.

Term	Gene count*	Genes	p-value	Bonferroni**	Benjamini**
**Immune response**	138	A.o.: BTK, CCL4, **CCR7**, CXCL16, CXCL8, CXCR5, **CX3CR1**, CLEC7A, CD14, CD1D, CD226, **CD28**, CD36, CD3D, CD58, CD79A, CD86, CD8B, CRK, FYN, FAS, FCER genes, JAK3, MYB, NLRP3, RAB29, REL, S100 genes, TAB2, **XIAP**, XAF1, AIF1, APOBEC genes, BTN3A3, CAMK4, CR1, CR2, C5AR1, CFP, CYBB, DLL1, EGR1, FCN1, FOXP1, HSP genes, HAVCR2, IFIT, IRF, IRAK2, IL23A, IL27RA, IL6R, KLRD1, LILRA genes, LY86, LY96, LEF1, LTB, HLA-DRA, HLA-DRB4, MAPK1, MAP3K1, NCF, NFKB, PRF1, PIK3R1, PF4, PARP9, STAT1, TLR2, TFEB, TRIM genes, TNFSF13B, TNFSF8	3.9E-19	2.5E-15	2.5E-15
**Defense response**	129	A.o.: ALS2, ANKHD, APOBEC genes, BTK, **CCL4**, CCR7, CXCL16, **CX3CR1**, CLEC7A, CD14, CD1D, CD226, **CD28**, CD36, CD58, CD86, CD8B, DDX3X, DDX60, FYN, FAS, FCER genes, IFI30, JAK3, KLF4, NLRP3, PRDM1, REL, S100 genes, TAB2, **XIAP**, XAF1, AIF1, ALOX5, CAMK4, **CASP1**, CSF3R, CR1, CR2, C5AR1, CFD, CFP, CST3, CYBB, EGR1, FCN1, FOXP1, FPR1, GFI1, HSP genes, HAVCR2, IG genes, IFNGR2, **IFNG**, IFIT genes, IRF genes, IL23A, IL27RA, IL6R, KLRD1, LILRA genes, LY genes, LYZ, HLA-DRA, HLA-DRB4, NCF genes, NFKB genes, PRF1, STAT1, TLR2	5.3E-13	3.4E-9	1.1E-9
**Cell activation**	87	A.o.: ABAT, ADAM10, BTK, **CCR7**, CXCL8, CXCR5, **CX3CR1**, CLEC7A, CD1D, CD2, CD226, **CD28**, CD3D, CD5, CD79A, CD86, CD8B, LIG4, FYN, FAS, FCER genes, JAK3, MAFB, MYB, NLRP3, SOX4, CAMK4, CR2, CST3, DLL1, EGR1, FOXP1, HAVCR2, IRS2, **IFNG**, IRF1, IL23A, IL27RA, IL6ST, LEF1, HLA-DRA, HLA-DRB4, PRF1, PIK3R1, PF4, SLAMF1, TLR2, TNFSF13B, TNFSF8, VCL	4.9E-12	3.2E-8	6.3E-9
**Regulation of immune system process**	116	A.o.: ADAM10, BTK, CCL4, **CCR7**, CXCL8, CLEC7A, CD14, CD1D, CD2, CD226, **CD28**, CD36, CD3D, CD5, CD79A, CD86, CD8B, CRK, DDX60, FYN, FANCL, FAS, FCER genes, JAK3, MAFB, MYB, RAB29, TAB2, **XIAP**, AIF1, CSF3R, CR1, CR2, CFD, CFP, DLL1, FCN1, FOXP1, FPR1, HSP genes, HAVCR2, IFNGR2, **IFNG**, IFIT1, IRF1, IRAK2, IL23A, IL27RA, IL6R, KLRD1, LILRB genes, LEF1, HLA-DRA, HLA-DRB4, MAPK1, MAP3K1, NFKB1, PIK3R1, PARP9, SLAMF1, TLR2, TNFSF13B, VEGFA	2.5E-11	1.6E-7	2.3E-8
**Leukocyte activation**	74	A.o.: ADAM10, BTK, **CCR7**, CXCL8, CXCR5, **CX3CR1**, CLEC7A, CD1D, CD2, CD226, **CD28**, CD3D, CD5, CD79A, CD86, CD8B, LIG4, FYN, FAS, JAK3, MAFB, MYB, RAB29, AIF1, SOX4, DLL1, FOXP1, HAVCR2, IRS2, **IFNG**, IRF1, IL23A, IL27RA, IL6ST, LEF1, HLA-DRA, HLA-DRB4, PRF1, PIK3R1,	3.9E-11	2.5E-7	3.2E-8
**Response to stress**	238	A.o.: ABAT, ALS2, APC, APOBEC genes, BTK, BCLAF1, CCL4, **CCR7**, CXCL16, CXCL8, **CX3CR1**, CLEC7A, CFLAR, CD14, CD1D, CD226, **CD28**, CD36, CD58, CD86, CD8B, DDX3X, DDX60, LIG4, POLH, FYN, FANCL, FANCM, FAS, JAK3, KLF4, MAFF, NLRP3, PRDM1, REL, S100 genes, SOX4, TIAM1, TAB2, **XIAP**, XAF1, ALOX5, CASP1, CDC7, CSF3R, CR1, CR2, C5AR1, CFD, CFP, EGR1, FCN1, FOXP1, FPR1, HSP genes, HAVCR2, **ID3**, IFNGR2, **IFNG**, IFIT genes, IRF1, IRF2, IL23A, IL27RA, IL6ST, LILRA genes, LYZ, HLA-DRA, HLA-DRB4, NCF genes, NFKB genes, PARP9, STAT1, SLAMF1, PIK3R1, PRF1, USP genes, VEGFA, TNFSF8	6.0E-11	3.8E-7	44.3E-8
**Cytokine production**	65	A.o.: BTK, **CCR7**, CD14, CD2, CD226, **CD28**, CD36, CD58, CD86, DDX3X, DDX60, JAK3, KLF4, MAF, NLRP3, REL, S100 genes, TIA1, CAMK4, **CASP1**, CYBB, DLL1, EGR1, FCN1, FOXP1, GBP1, HSP genes, HAVCR2, IFI16, **IFNG**, IRF1, IL23A, IL27RA, IL6R, IL6ST, LEF1, LY96, LTB, NFKB1, NFKB2, HLA-DRB4, PF4, SLAMF1, TLR2	2.3E-10	1.5E-6	1.5E-7
**T cell activation**	50	A.o.: **CCR7**, CLEC7A, CD1D, CD2, **CD28**, CD3D, CD5, CD85, CD8B, LIG4,FYN, FAS, FCER1G, JAK3, MAFB, MYB, NLRP3, PRDM1, RAB29, SOX4, AIF1, BATF1, CAMK4, EGR1, FOXP1, HAVCR2, ITPKB, **IFNG**, IFR1, IL23A, IL6ST, LEF1, HLA-DRA, HLA-DRB4, PIK3R1, PRNP, RHOH, SLAMF1, TNFSF13B, TNFSF8	3.6E-9	2.3E-5	1.2E-6

*Total 1024 differentially expressed genes in LGL versus TemRA dataset with FC = 2 both up- and down-regulated, p<0.05 (ANOVA), of which 805 were annotated by DAVID using Affymetrix Human Genome U133 Plus 2.0 array as background and selecting Homo Sapiens as species.

**Adjusted p-value based on Bonferroni and Benjamini-Hochberg correction for multiple testing.

Genes also identified through IPA analyses, which are further validated with RQ-PCR are indicated in bold.

**Table 3:** KEGG enrichment pathway analysis of TCRγδ+ T-LGL leukemia cases versus healthy TCRγδ+ TemRA cells in DAVID.

Term	Gene count	Genes	p-value	Bonferroni*	Benjamini*
**Hematopoietic cell lineage**	16	CD14, CD1d, CD2, CD36, CD3d, CD5, CD8b, ANPEP, CSF3R, CR1, CR2, ITGA6, IL6R, HLA-DRA, HLA-DRB, MS4A1	1.3E-5	3.3E-3	3.3E-3
**Osteoclast differentiation**	19	BTK, FYN, TAB2, CAMK4, CYBB, IFNGR2, **IFNG**, LILRA/LILRB genes, NCF1, NCF2, NFKB1, NFKB2, PIK3R1, SIRPA, STAT1	9.9E-5	2.6E-2	1.3E-2
**Rheumatoid arthritis**	13	ATP6V genes, CXCL8, **CD28**, CD86, **IFNG**, IL23A, LTB, HLA-DRA, HLA-DRB4, TLR2, TNFSF13B, VEGFA	8.1E-4	1.9E-1	5.2E-2
**NF-kappa B signaling pathway**	13	BTK, CCL4, CXCL8, **CFLAR**, CD14, TAB2, **XIAP**, LY96, LTB, NFKB1, NFKB2, PIAS4, TNFSF13B	1.0E-2	2.3E-1	5.1E-2

*Adjusted p-value based on Bonferroni and Benjamini-Hochberg corrections for multiple testing.

Genes also identified through IPA analyses, which are further validated with RQ-PCR, are indicated in bold.

**Fig 2:**
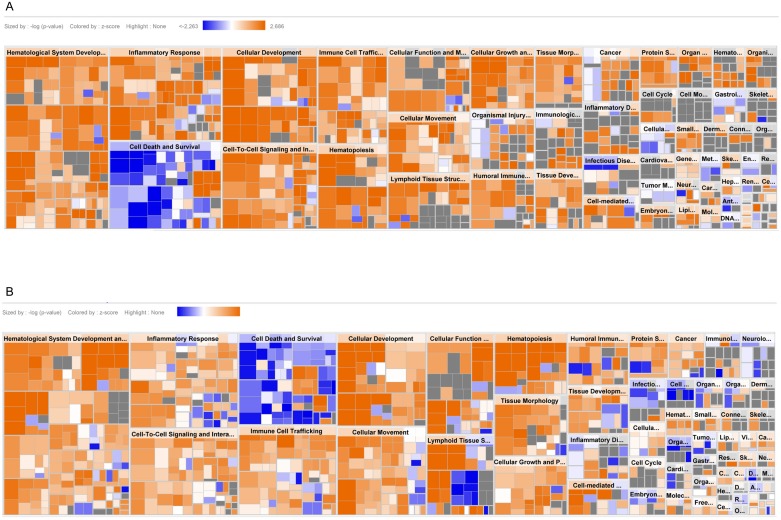
Functional annotation of genes differentially expressed between TCRγδ+ T-LGL leukemia cases and healthy control TCRγδ+ T cell subsets. (A) Comparison of TCRγδ+ T-LGL leukemia cases versus healthy control TemRA and (B) TemRO TCRγδ+ T cell subsets showing similar patterns with down-regulation of apoptosis and up-regulation of cancer-related processes. Sizing based on significance level (ANOVA), up- and down-regulation based on z-scoring. Plots were generated in QIAGEN’s Ingenuity Pathway Analysis.

For further interpretation and visualization of differentially expressed genes, Ingenuity Pathway Analysis (IPA) was used. First, TCRγδ+ T-LGL leukemia cases were compared to healthy TemRA TCRγδ+ T cells, showing a number of processes which were up-regulated in the TCRγδ+ T-LGL leukemia cases, such as hematological development and function. However, cell death and survival processes appeared to be significantly down-regulated (Fig 2A). Again, similar results were obtained after analyzing the differentially expressed probe sets between TCRγδ+ T-LGL leukemia cases and the healthy TemRO subset (Fig 2B), confirming comparable correlation of both subsets with the TCRγδ+ T-LGL leukemias as described from the unsupervised analysis (Fig 1).

After identifying affected processes we focused more in-depth on specific genes. Genes involved in apoptosis, such as *BCLAF1*, *CASP1*, *XIAP* and *CFLAR*, appeared to be the most relevant candidate genes. The apoptosis-inducing genes *CASP1* and *CFLAR* were down-regulated, as well as the apoptosis inhibitor *XIAP*, while *BCLAF1* was up-regulated. Furthermore, transcription factors such as *ID3*, *KLF4*, *LEF1* and *SOX4* in the up-regulated cell survival process were all found to be up-regulated. Finally, altered expressions of *CD28*, *CCR7*, *CX3CR1*, *IFNG*, *LTB* and *PRF1* were all contributing to the inflammatory profile of the TCRγδ+ T-LGL leukemias.

Collectively, data from all analysis methods (DAVID Gene Ontology, DAVID KEGG, IPA) thus showed that proliferation and cell survival signaling were up-regulated, while apoptosis was down-regulated in TCRγδ+ T-LGL leukemia cells when compared to normal TCRγδ+ T cell subsets.

### RQ-PCR validation indicates possible signature genes within a highly heterogeneous disease profile

**Fig 3:**
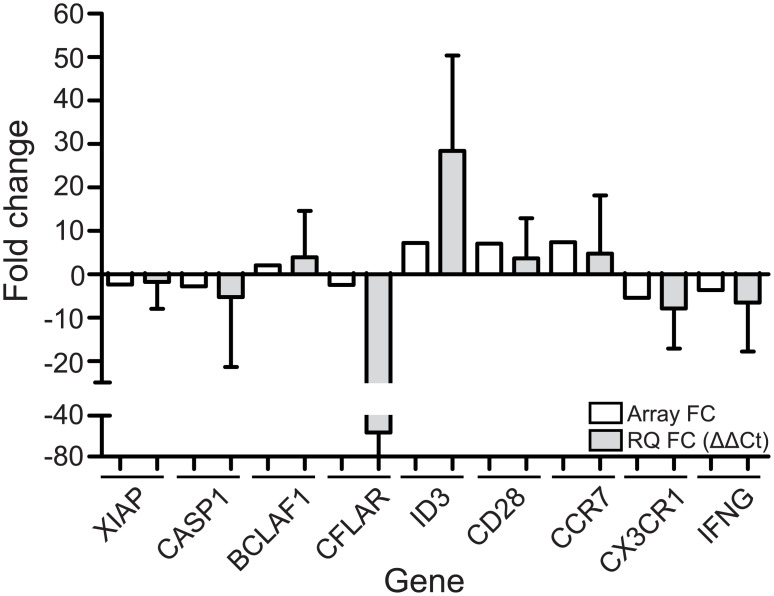
RQ-PCR validation of most representative genes. Fold changes of representative genes identified through gene expression profiling and by RQ-PCR. Relative expression of genes found differentially expressed using microarrays was first normalized to ABL housekeeping gene (ΔCt). Average ΔCt values from healthy controls (N = 6) were used to calculate patient (N = 10) to healthy control ratios per patient to obtain fold change values (ΔΔCt). White bars depict fold change values obtained from microarray data, grey bars depict average ΔΔCt values after RQ-PCR validation. For RQ-PCR validation 4 patients from the original microarray dataset were used and 6 novel patients. Mean expression fold change values are indicated with the standard deviation.

Next, representative candidate genes, which were found in the comparison between TCRγδ+ T-LGL leukemia cells and healthy TemRA and TemRO TCRγδ+ T cells in both IPA and DAVID analysis, and which appeared biologically relevant for the disease, were selected. These included genes involved in cell death and survival processes (*BCLAF1*, *CFLAR*, *CASP1*, and *XIAP*), genes associated with proliferation-driving transcription or cancer (*KLF4*, *LEF1*, *SOX4*, *ID3*), and genes associated with inflammation (*CD28*, *CCR7*, *CX3CR1* and *IFNG*). Differences in gene expression levels were analyzed by means of real-time quantitative (RQ)-PCR using Universal Probe Library reagents. Sorted healthy control TemRA TCRγδ+ T cells were compared with the sorted TCRγδ+ T-LGL leukemia cells from patients (both from patients included in the microarray analysis, and from new patients). In order to validate the fold changes obtained from microarray analysis, expression ratios between patients and healthy controls were calculated from the RQ-PCR data. All selected apoptosis-related genes from our microarray analysis, were confirmed to be differentially expressed in TCRγδ+ T-LGL leukemia cases when compared to healthy controls; *XIAP*, *CASP1*, *BCLAF1* were all validated to be over two-fold higher, and especially *CFLAR* to an even higher extent than observed in the microarrays (Fig 3). Transcription factor *ID3* could also be validated in RQ-PCR; the ratio between TCRγδ+ T-LGL leukemia patients and healthy controls was even higher than that observed in the microarrays. Other transcription factors identified from the microarray analysis (*KLF4*, *LEF1* and *SOX4*) could not be confirmed by RQ-PCR validation (S6 Table). *CD28*, *CCR7* and *CX3CR1* were validated as well, albeit with a lower fold change than in the microarrays. *IFNG* was validated with an approximately two-fold higher fold change in RQ-PCR than in the microarrays (Fig 3). Of note, considerable heterogeneity in RQ-PCR levels was observed within the samples used for validation (S6 Table), thus reflecting the heterogeneity of the disease.

Taken together, we were able to identify possible candidate genes to distinguish aberrant from normal TCRγδ+ T cells, although the known largely heterogeneous profile of the disease remains noticeable, which may partly mask differences between leukemic TCRγδ+ T-LGL cells and normal TCRγδ+ T cell subsets.

## Discussion

T cell large granular lymphocyte disorders form a large group of heterogeneous mature T cell neoplasms, of which the TCRγδ+ T cell variant is rare and most poorly understood. TCRγδ+ T-LGL leukemia is known to have a slowly progressive indolent disease course, mostly affecting elderly individuals, while showing similar (initial) clinical presentations such as neutropenia, anemia and pancytopenia [4,6,25]. Most of our patients displayed malignancy, autoimmunity or cytopenia as underlying or associated diseases, as described previously [26], but there was no consistent pattern. Furthermore, immunophenotypic features did not correlate with the symptomatic status or underlying diseases. Underlying (chronic) diseases could play a role in the pathogenesis of TCRγδ+ T-LGL leukemia, possibly as a (chronic) stimulus that causes the TCRγδ+ T cells to expand, in line with the idea that TCRγδ+ T-LGL leukemia develops from dominant normal TCRγδ+ T cell populations in the adult peripheral blood. However, underlying molecular mechanisms in TCRγδ+ T-LGL leukemogenesis are largely unknown, and a common underlying molecular defect has not been described earlier.

TCRγδ+ T-LGL leukemias are characterized by skewed receptor expression and an antigen stimulation-associated activation status, but are also considered to harbour particular changes in gene expression. In our current study we aimed to address this by means of gene expression profiling, which revealed that the TCRγδ+ T-LGL leukemia cells displayed different gene expression profiles when compared to healthy, polyclonally expanded TCRγδ+ T cells. Initial unsupervised clustering based on microarray data from aberrant TCRγδ+ T cells and different healthy TCRγδ+ T cell subsets, showed a clear distinction between the TCRγδ+ T-LGL leukemia and its healthy counterparts. Even though TCRγδ+ T-LGL leukemia forms a separate group, with the lowest correlation with the naive subset as expected, the correlation with the TemRA subset was quite high, suggesting that it is derived from the most predominant and antigen-experienced type of TCRγδ+ T cell populations in adult peripheral blood, in line with earlier published data [27]. When focusing more in-depth on differences in comparison with normal TemRA TCRγδ+ T cells it became clear that particular biological functions, processes and genes were affected. Most relevant genes involved processes such as proliferation, stimulation and apoptosis, with an increase in proliferation and cell survival, and a decrease in apoptosis. One clear example concerned transcription factor *ID3*, which is involved in proliferation and haematological development, a.o. by enhancing TCRγδ+ T cell development [28]. Up-regulation in TCRγδ+ T-LGL leukemia cases could be confirmed with RQ-PCR. As opposed to proliferation genes, some apoptosis-inducing genes such as *CASP1* and *CFLAR* were down-regulated. Caspase-1 is an apoptosis-related cysteine peptidase, which is involved in inflammation and apoptosis by proteolysis of pro-inflammatory cytokines and activating other caspases and pro-apoptotic proteins [29,30]. *CFLAR* has been described in multiple studies as being important in the development of cancer, and is therefore also being described as a target for therapy (reviewed by Fulda in 2013 [31]); it induces apoptosis [32], but is also a key role player in autophagy and necroptosis [33]. *XIAP*, which normally regulates and inhibits apoptosis [34] was also shown to be down-regulated, resulting in a net effect of inhibited apoptosis. The up-regulated apoptosis-related gene *BCLAF1* induces apoptosis and represses transcription [35]. Our RQ-PCR validation data also showed up-regulation of *BCLAF1*, and down-regulation of *CFLAR* and *CASP1*. All apoptosis-related genes could be confirmed with RQ-PCR, thus showing consistency of these shared aberrancies in different TCRγδ+ T-LGL leukemia patients. Interestingly, our data on TCRγδ+ T-LGL leukemia are thus completely in line with earlier studies, showing signaling towards survival through positive regulation of T cell receptor signaling and an enhanced immune response, rather than towards induction of apoptosis [36,37]. Additionally, the supervised analysis yielded aberrancies in the normal functioning of the TCRγδ+ T-LGL lymphoproliferative cells, with more skewing towards activation and inflammation given the up-regulation of *CD28* and *CCR7*. *IFNG* on the other hand was down-regulated, implying that normal functioning of TCRγδ+ T cells through IFN-γ production upon activation during infection [38], is lost. These expression levels were also confirmed on fold change level by means of RQ-PCR. Furthermore, communication of the TCRγδ+ T-LGL leukemia cells with other immune cells was affected as reflected by the down-regulation of chemokine *CX3CR1*.

In CD8+ TCRαβ+ T-LGL leukemia altered signaling through STATs has been implicated in the leukemogenesis, based on the frequent occurrence in mutations in the STAT3 and/or STAT5b genes [39,40]. In our cohort of 10 TCRγδ+ T-LGL leukemia cases analyzed with gene expression profiling we checked expression levels of STAT3 and STAT5b genes but did not find significant alterations as compared to healthy control TCRγδ+ T cell subsets. This might suggest that STAT3 and STAT5b are less clearly implicated in the pathogenesis of TCRγδ+ T-LGL leukemia than CD8+TCRαβ+ T-LGL and/or NK-LGL leukemia. Furthermore, it has been shown previously that the survival of leukemic T-LGL cells is rather through STAT3-independent signaling [36].

Notably, not all genes could be confirmed with the same fold change in microarrays and RQ-PCR. High heterogeneity in expression levels within both healthy controls and patients was observed. This could be due to general differences between individuals, displaying different gene expression profiles, ranging from ones with high proliferative activity of TemRA TCRγδ+ T cells, to ones with low activity. Therefore, a more extended investigation into the aberrant apoptotic profile of a higher number of TCRγδ+ T-LGL leukemia patients is warranted. Furthermore, stimulation of the TCRγδ+ T-LGL leukemia cells, or *in vitro* blocking of the apoptotic genes identified and validated in our study, should provide more insights in the activation profile or the activity against for instance other blood cells, which could potentially explain the accompanying cytopenia as seen in these patients. Also, this could shed light on the underlying stimulations of the TCRγδ+ T cells required for proliferation. Additionally, novel techniques such as RNA-sequencing might be helpful in creating a broader perspective on the disease, including more information about possible splice variants and single nucleotide variants.

## Conclusion

Overall, our current study provides more insight in the pathogenesis of TCRγδ+ T-LGL leukemia by showing a disturbed balance in proliferation and apoptosis, but also in immune and inflammatory responses and normal functioning of the TCRγδ+ T cells. TCRγδ+ T-LGL leukemia cells originate from antigen-experienced normal TemRA/TemRO TCRγδ+ T cells in adult peripheral blood, but these TCRγδ+ T-LGL cells have undergone a shift in the proliferation-apoptosis balance, towards increased proliferation and survival.

## Supporting information

S1 Fig Gating strategies for FACS-based sorting experiments for sorting patient and healthy control material. (A) General gating strategy to exclude debris and doublets, and to define lymphocytes based on CD45. (B) Further gating strategy for sorting patient material and TCRVδ1 and TCRVδ2 healthy subsets, and (C) gating strategy for sorting maturation subsets naive (CD45RA+CD45RO-CD197+CD27+), central memory (CD45RA-CD45RO+CD197+CD27+), TemRO (CD45RA-CD45RO+CD197-CD27-) and TemRA (CD45RA+CD45RO-CD197-CD27-).
(EPS)

S1 Table Antibody details for FACS-based cell sorting experiments. * Maturation subsets naive (Tn), central memory (Tcm), TemRO and TemRA cells. Naive cells are defined as CD45RA+CD45RO-CD197+CD27+, central memory cells as CD45RA-CD45RO+CD197+CD27+, TemRO cells as CD45RA-CD45RO+CD197-CD27- and TemRA cells as CD45RA+CD45RO-CD197-CD27-.
(DOCX)

S2 Table Primers and probes from Roche Universal Probe Library for RQ-PCR design. *Reverse complementary primers. **Probe numbers according to the Roche Universal Probe Library.
(DOCX)

S3 Table Differentially expressed probe sets between TCRγδ+ T-LGL leukemia cases and healthy TCRγδ+ T cell subsets after different supervised statistical analyses. *Two-way, multiple factor analysis of variance (ANOVA), p<0.05. **Significance analysis of microarrays (SAM), p<0.05.
(DOCX)

S4 Table Top up- and down-regulated genes after supervised analysis. Top 25 up- and down-regulated genes after supervised analysis on significance level of ANOVA p<0.05. *The adjusted p-value is after Benjamini-Hochberg correction for multiple testing. **The Fold Change is based on probe set intensity in LGL cases versus healthy control after normalization and statistical and multiple testing.
(DOCX)

S5 Table Gene ontology biological processes and KEGG enrichment pathway analysis of TCRγδ+ T-LGL leukemia cases versus healthy TCRγδ+ TemRO cells in DAVID. *Total 1686 differentially expressed genes in LGL versus TemRO dataset with FC = 2 both up- and down-regulated, p<0.05 (ANOVA), of which 1563 were annotated by DAVID using Affymetrix Human Genome U133 Plus 2.0 array as background and selecting Homo Sapiens as species. **Adjusted p-value based on Bonferroni and Benjamini-Hochberg correction for multiple testing. Genes also identified through DAVID LGL versus effector subset and IPA analyses, which are further validated with RQ-PCR are indicated in bold.
(DOCX)

S6 Table Median (interquartile range) mRNA expressions, median ΔΔCt and microarray fold changes of genes used for RQ-PCR validation. *Relative mRNA expression after *ABL* housekeeping gene correction. **Median ΔΔCt value of TCRγδ+ T-LGL leukemia patients after correction with average ΔCt values from healthy control samples. ***Fold changes according to supervised TCRγδ+ T-LGL leukemia cells vs. healthy TemRA TCRγδ+ T cells comparison.
(DOCX)
