# Supplementary material for: Dysregulated signaling, proliferation and apoptosis impact on the pathogenesis of TCRγδ+ T cell large granular lymphocyte leukemia
Source: PLoS One. 2017 Apr 13;12(4):e0175670. doi: 10.1371/journal.pone.0175670 (PMC5391076; doi:10.1371/journal.pone.0175670)
Supplement: S1 Table — * Maturation subsets naive (Tn), central memory (Tcm), TemRO and TemRA cells. Naive cells are defined as CD45RA+CD45RO-CD197+CD27+, central memory cells as CD45RA-CD45RO+CD197+CD27+, TemRO cells as CD45RA-CD45RO+CD197-CD27- and TemRA cells as CD45RA+CD45RO-CD197-CD27-. (DOCX) [file pone.0175670.s002.docx]

**S1 Table.** **Antibody details for FACS-based cell sorting experiments.**

| **Tube** | | **Fluorochrome** | | | | | | | | **Used for / applied on** |
| --- | --- | --- | --- | --- | --- | --- | --- | --- | --- | --- |
|  |  | **PB** | **PO** | **FITC** | **PerCP-Cy5.5** | **PE** | **PE-Cy7** | **APC** | **APC-H7** |  |
| **1** | **Antibody**  Clone  Manufacturer |  | **CD45**  HI30  Invitrogen | **TCRVδ1**  TS8.2  Thermo Scientific | **CD3**  SK7 BD Biosciences | **TCRVδ2**  B6  BD Biosciences | **CD19**  SJ25C1  BD Biosciences | **TCRαβ**  IP26  e-Bioscience |  | Healthy control Vδ1/Vδ2 subsets;  Patient material, sorting on tumor-specific cells |
| **2** | **Antibody**  Clone  Manufacturer | **CD3**  UCHT1  BD Biosciences | **CD45**  HI30  Invitrogen | **TCRαβ**  WT31  BD Biosciences | **CD27**  L128  BD Biosciences | **CD197**  3D13  e-Bioscience | **CD45RO**  UCHL1  BD Biosciences | **CD45RA**  HI100  BD Biosciences | **CD19**  SJ25C1  BD Biosciences | Healthy control, Tn, Tcm, TemRO and TemRA subsets* |

* Maturation subsets naive (Tn), central memory (Tcm), TemRO and TemRA cells. Naive cells are defined as CD45RA+CD45RO-CD197+CD27+, central memory cells as CD45RA-CD45RO+CD197+CD27+, TemRO cells as CD45RA-CD45RO+CD197-CD27- and TemRA cells as CD45RA+CD45RO-CD197-CD27-.
