# Supplementary material for: Dysregulated signaling, proliferation and apoptosis impact on the pathogenesis of TCRγδ+ T cell large granular lymphocyte leukemia
Source: PLoS One. 2017 Apr 13;12(4):e0175670. doi: 10.1371/journal.pone.0175670 (PMC5391076; doi:10.1371/journal.pone.0175670)
Supplement: S2 Table — *Reverse complementary primers. **Probe numbers according to the Roche Universal Probe Library. (DOCX) [file pone.0175670.s003.docx]

**S2 Table.** **Primers and probes from Roche Universal Probe Library for RQ-PCR design.**

| **Gene** | **Forward** | **Reverse*** | **Probe**** |
| --- | --- | --- | --- |
| BCLAF1 | AGTCTAGGGGCCGTTCCTC | TCCCAGTCTTTGCAGTTTCC | 22 |
| CASP1 | TCACTGCTTCGGACATGACT | GCTGTCAGAGGTCTTGTGCTC | 53 |
| CCR7 | GCTCAAGACCATGACCGATAC | CAGAAGGGAAGGGTCAGGA | 87 |
| CD28 | CTAAGCCCTTTTGGGTGCT | CCAGAAAATAATAAAGGCCACTG | 22 |
| CFLAR | CTCAGGAACCCTCACCTTGT | CAGATTTATCCAAATCCTCACCA | 53 |
| CX3CR1 | CAGTGACAGAAAACTTTGAGTACGA | AGACCACGATGTCCCCAATA | 30 |
| FAS | ATGGCCAATTCTGCCATAAG | TGACTGTGCAGTCCCTAGCTT | 65 |
| ID3 | CCTGTCGGAACGCAGTCT | ATGTCGTCCAGCAAGCTCA | 73 |
| IFNG | GGAAAGAGGAGAGTGACAGAAAA | TTGGATGCTCTGGTCATCTTTA | 21 |
| KLF4 | GGGAGAAGACACTGCGTCA | GGAAGCACTGGGGGAAGT | 52 |
| LEF1 | CCAAACAAGGCATGTCCA | CCGGAGACAAGGGATAAAAAG | 88 |
| LTB | CTTCTCTGGTGACCTTGTTGC | CCTGATCCTGGGGCACTA | 76 |
| PRF1 | GAGGGTGTGGACGTGACC | CAGGAACCTTTGTGTGTCCA | 12 |
| SOX4 | CAACGCCGAGATCTCCA | GGATCTTGTCGCTGTCTTTGA | 11 |
| XIAP | TGGTATCCAGAATGGTCAGTACA | TGGCCTGTCTAAGGCAAAAT | 38 |

*Reverse complementary primers.

**Probe numbers according to the Roche Universal Probe Library.
