# Supplementary material for: Dysregulated signaling, proliferation and apoptosis impact on the pathogenesis of TCRγδ+ T cell large granular lymphocyte leukemia
Source: PLoS One. 2017 Apr 13;12(4):e0175670. doi: 10.1371/journal.pone.0175670 (PMC5391076; doi:10.1371/journal.pone.0175670)
Supplement: S3 Table — *Two-way, multiple factor analysis of variance (ANOVA), p<0.05. **Significance analysis of microarrays (SAM), p<0.05. (DOCX) [file pone.0175670.s004.docx]

**S3 Table. Differentially expressed probe sets between TCRγδ+ T-LGL leukemia cases and healthy TCRγδ+ T cell subsets after different supervised statistical analyses.**

| **TCRγδ+ T-LGL leukemia vs. subset** | **Statistical level** | **Total differentially expressed probe sets** | **Up-regulated** | **Down-regulated** |
| --- | --- | --- | --- | --- |
| Naive | ANOVA 0.05* | 8016 | 3037 | 4979 |
|  | SAM 0.05** | 16199 | 13027 | 3172 |
| Effector | ANOVA 0.05 | 1633 | 681 | 952 |
|  | SAM 0.05 | 298 | 298 | 0 |
| Effector memory | ANOVA 0.05 | 2653 | 1222 | 1431 |
|  | SAM 0.05 | 513 | 506 | 7 |

*Two-way, multiple factor analysis of variance (ANOVA), p<0.05.

**Significance analysis of microarrays (SAM), p<0.05.
