# Supplementary material for: Dysregulated signaling, proliferation and apoptosis impact on the pathogenesis of TCRγδ+ T cell large granular lymphocyte leukemia
Source: PLoS One. 2017 Apr 13;12(4):e0175670. doi: 10.1371/journal.pone.0175670 (PMC5391076; doi:10.1371/journal.pone.0175670)
Supplement: S4 Table — Top 25 up- and down-regulated genes after supervised analysis on significance level of ANOVA p<0.05. *The adjusted p-value is after Benjamini-Hochberg correction for multiple testing. **The Fold Change is based on probe set intensity in LGL cases versus healthy control after normalization and statistical and multiple testing. (DOCX) [file pone.0175670.s005.docx]

**S4 Table. Top up- and down-regulated genes after supervised analysis.**

| **Gene symbol** | **Gene name** | **Adjusted p-value*** | **Fold Change**** |
| --- | --- | --- | --- |
| *Top 25 up-regulated genes in TCRγδ+ T-LGL leukemia cases relative to effector cells* | | | |
| LYZ | Lysozyme | 0.000 | 36.170 |
| S100A8 | S100 calcium binding protein A8 | 0.000 | 26.823 |
| VCAN | Versican | 0.000 | 19.917 |
| S100A9 | S100 calcium binding protein A9 | 0.000 | 18.571 |
| RCAN3 | RCAN family member 3 | 0.000 | 18.062 |
| FCN1 | Ficolin 1 | 0.000 | 16.518 |
| G0S2 | G0/G1 switch 2 | 0.000 | 15.844 |
| NELL2 | Neural EGFL like 2 | 0.000 | 15.712 |
| CSTA | Cystatin A | 0.000 | 14.029 |
| SERPINB6 | Serpin family B member 6 | 0.000 | 13.542 |
| SOX4 | SRY (sex determining region Y) box 4 | 0.000 | 13.277 |
| MAFB | v-maf avian musculoaponeurotic fibrosarcoma oncogene homolog | 0.000 | 12.790 |
| CPVL | Carboxypeptidase, vitellogenic like | 0.000 | 11.861 |
| IRS2 | Insulin receptor substrate 2 | 0.000 | 11.489 |
| ALOX5 | Arachidonate 5-lipoxygenase | 0.000 | 11.421 |
| GPR183 | G protein-coupled receptor 183 | 0.000 | 10.627 |
| MAL | Mal T cell differentiation protein | 0.000 | 9.505 |
| TGFBI | Transforming growth factor beta induced | 0.000 | 9.118 |
| CD36 | CD36 molecule | 0.000 | 8.746 |
| CLEC7A | C-type lectin domain family 7 member A | 0.000 | 8.647 |
| PLBD1 | Phospholipase B domain containing 1 | 0.000 | 8.415 |
| SCML1 | Sex comb on midleg-like 1 (Drosophila) | 0.000 | 8.397 |
| LTB | Lymphotoxin beta | 0.000 | 7.338 |
| CCR7 | C-C motif chemokine receptor 7 | 0.000 | 7.377 |
| AIF1 | Allograft inflammatory factor 1 | 0.000 | 7.290 |
| *Top 25 down-regulated genes in TCRγδ+ T-LGL leukemia cases relative to effector cells* | | | |
| ENPP5 | Ectonucleotide pyrophosphatase/phosphodiesterase 5 | 0.000 | -6.938 |
| HS3ST3B1 | Heparan sulfate-glycosamine 3-sulfotransferase 3B1 | 0.000 | -6.191 |
| ST8SIA4 | ST8 alpha-N-acetyl-neuraminide alpha-2,8-sialyltransferase 4 | 0.000 | -6.137 |
| ETNK1 | Ethanolamine kinase 1 | 0.000 | -5.674 |
| IFIT3 | Interferon induced protein with tetratricopeptide repeats 3 | 0.000 | -5.458 |
| CX3CR1 | C-X3-C motif chemokine receptor 1 | 0.000 | -5.443 |
| PCMT1 | Protein-L-isoaspartate (D-aspartate) O-methyltransferase | 0.000 | -4.934 |
| SNTB2 | Syntrophin beta 2 | 0.000 | -4.769 |
| ELOVL6 | ELOVL fatty acid elongase 6 | 0.000 | -4.399 |
| PRR5L | Proline rich 5 like | 0.000 | -4.390 |
| CD226 | CD226 molecule | 0.000 | -4.369 |
| PRF1 | Perforin 1 | 0.000 | -4.238 |
| ZNF260 | Zinc finger protein 260 | 0.000 | -4.094 |
| MAN1A1 | Mannosidase alpha class 1A member 1 | 0.000 | -4.087 |
| DYNLL1 | Dynein light chain LC8-type 1 | 0.000 | -4.061 |
| TOMM5 | Translocase of outer mitochondrial membrane 5 | 0.000 | -3.989 |
| CTBP2 | C-terminal binding protein 2 | 0.000 | -3.938 |
| G3BP1 | G3BP stress granule assembly factor 1 | 0.000 | -3.918 |
| KATNBL1 | Katanin regulatory subunit B1 like 1 | 0.000 | -3.913 |
| RASGEF1A | RasGEF domain family member 1A | 0.000 | -3.896 |
| GTF2H5 | General transcription factor IIH subunit 5 | 0.000 | -3.853 |
| SKA2 | Spindle and kinetochore associated complex subunit 2 | 0.000 | -3.825 |
| PEX3 | Peroxisomal biogenesis factor 3 | 0.000 | -3.815 |
| ERMP1 | Endoplasmic reticulum metallopeptidase 1 | 0.000 | -3.807 |
| ARFIP1 | ADP ribosylation factor interacting protein 1 | 0.000 | -3.781 |

Top 25 up- and down-regulated genes after supervised analysis on significance level of ANOVA p<0.05. *The adjusted p-value is after Benjamini-Hochberg correction for multiple testing.
**The Fold Change is based on probe set intensity in LGL cases versus healthy control after normalization and statistical and multiple testing.
