# Supplementary material for: Dysregulated signaling, proliferation and apoptosis impact on the pathogenesis of TCRγδ+ T cell large granular lymphocyte leukemia
Source: PLoS One. 2017 Apr 13;12(4):e0175670. doi: 10.1371/journal.pone.0175670 (PMC5391076; doi:10.1371/journal.pone.0175670)
Supplement: S5 Table — *Total 1686 differentially expressed genes in LGL versus TemRO dataset with FC = 2 both up- and down-regulated, p<0.05 (ANOVA), of which 1563 were annotated by DAVID using Affymetrix Human Genome U133 Plus 2.0 array as background and selecting Homo Sapiens as species. **Adjusted p-value based on Bonferroni and Benjamini-Hochberg correction for multiple testing. Genes also identified through DAVID LGL versus effector subset and IPA analyses, which are further validated with RQ-PCR are indicated in bold. (DOCX) [file pone.0175670.s006.docx]

**S5 Table. Gene Ontology biological processes and KEGG enrichment pathway analysis of TCRγδ+ T-LGL leukemia cases versus healthy TCRγδ+ TemRO cells in DAVID.**

| **Gene Ontology biological processes** | | | | | |
| --- | --- | --- | --- | --- | --- |
| **Term** | **Gene count*** | **Genes** | **p-value** | **Bonferroni**** | **Benjamini**** |
| **Adaptive immune response** | 29 | CLEC10A, CD244, CD6, CD86, CD8B, DCLRE1C, GPR183, KLRC1-KLRK1, PRDM1, SH2D1A, SLAMF7, TRAT1, ALCAM, ADGRE1, CRACR2A, CAMK4, CTLA4, EOMES, HAVCR2, IGHM, **IFNG**, LILRA2/B2/B3, LAX1, LAMP3, PIK3CG, PAG1, TFEB | 9.8E-8 | 3.7E-4 | 3.7E-4 |
| **Neutrophil chemotaxis** | 17 | CCL3L3, CCL4, CXCL8, CXCR2, FCER1F, S100A12/8/9, CSF3R, C5AR1, **IFNG**, JAML, PIK3CG, PDE4B/D, PPBP, VAV3 | 1.2E-6 | 4.3E-3 | 2.2E-3 |
| **Innate immune response** | 50 | ANKHD1, APOBEC3A_B, CLEC10A/7A, CD14, CD1D, CD244, CD6, DDX3X, DDX58/60, FGR, FCER1G, KLRC4-KLRK1, NLRP3, PRDM1, REL, S100A12/8/9, SH2D1A, SRPK2, APP, APOBEC3F/G, CR1, CYBB, HAVCR2, IGHM, IFI16, IL23A, KIR3DS1, KIR2DS1/2/5, KLRD1, LILRA5, LY86/96, MID2, NCF1/2, NFKB2, PTX3, PIK3CG, RNF135, SLAMF1, SUSD4, TLR2 | 9.2E-6 | 3.4E-2 | 1.1E-2 |
| **Cellular defense response** | 15 | CCR6, CXCR2, **CX3CR1**, SH2D1A, TRAT, C5AR1, KLRC3/4, LILRB2, LY96, MNDA, NCR1, NCF1/2, PRF1 | 1.5E-5 | 5.4E-2 | 1.4E-2 |
| **Cellular response to DNA damage stimulus** | 32 | APC, BCL6, FANCL, MRE11, NEK4, SETD7, SLF2, SHPRH, SMARCAL1, **XIAP**, ARMT1, CHD2, CDKN1A, CTLA4, CTX3L, FOXO1, MCM8, MAPK1, MNDA, NFATC, PLK3, PSEN1, SETX, SGK1, SMC6, UBR5, USP28/47, MYC, VAV3, ZBTB38, ZMAT3 | 1.9E-5 | 6.9E-2 | 1.4E-2 |
| **Immune response** | 48 | CCL4, CCR6, **CCR7**, CXCL8, CXCR5, CD1D, CD27, CD36, CD86. CD8B, FCGR3A/B, GPR183, LY75-CD302, PRELID1, CTSC, C5AR1, CFP, CTLA4, FTH1, GZMH, ICOS, **IFNG**, IFITM2, IL27RA, KIR3DL and KIR2DL genes, LILRB2, LST1, LAX1, LTB, MARCH1, MBP, NCF1, OSM, PF4, PPBP, PRKRA, TLR2, TCF7, TNFSF13B/8, ZEB1 | 2.2E-5 | 8.0E-2 | 1.4E-2 |
| **Apoptotic process** | 56 | DHCR2, **BCLAF1**, **CFLAR**, CD14, CFFB, FAIM, GRAMD4, NCKAP1, NLRP3, NME6, PRELID1, POLR2G, S100A8/9, TIAM1, TIA1, **XIAP**, XAF1, APAF1, ARRB1, AHR, BEX2, **CASP1**, CTSC, C5AR1, CST3, CSRNP1, CIAPIN1, DAXX, DYNLL, ECE1, EFNA5, FEM1B, FOXO1, GZMB/H, GADD45A/B, ING4, **IFNG**, IRF, LY86, MAL, MAPK1, MAP3K1, NCF1, PPID, PRF1, PLK3, PPP2R2B, RPS6KA1, SGK1, STAT1, TLR2, TGFBR1, TNFSF8 | 1.5E-3 | 1.0E0 | 4.4E-1 |
| **KEGG enrichment pathway analysis** | | | | | |
| **Term** | **Gene count*** | **Genes** | **p-value** | **Bonferroni**** | **Benjamini**** |
| **Antigen processing and presentation** | 19 | CD8B, IFI30, HSP90AB1, HSPA6, **IFNG**, KIR3DL and KIR2DL/DS genes, KLRC3/4, KLRD1, NFYB, RFXAP | 2.2E-6 | 5.9E-4 | 5.9E-4 |
| **Natural killer cell mediated cytotoxicity** | 25 | CD244, FCER1G, FCGR3A/B, KLRC4-KLRK1, SH2D1A, GZMB, IFNGR2, **IFNG**, KIR2DL and KIR2DS genes, KLRD1, MAPK1, NCR1, NFATC2, PRF1, PIK3CG, PIK3R1, RAC2, VAV3 | 5.0E-6 | 1.3E-3 | 6.7E-4 |
| **Osteoclast differentiation** | 22 | FCGR3A/B, CAMK4, CYBB, IFNGR2, **IFNG**, LILRA/B genes, MAPK1, NCF1/2/4, NFKB2, NFATC2, PIK3CG, PIK3R1, SIRPA, STAT1, SOCS3, TGFBR1 | 7.5E-4 | 1.8E-1 | 6.5E-2 |
| **Rheumatoid arthritis** | 15 | ATPase genes, CCL3L3, CXC8, **CD28**, CD86, CTLA4, **IFNG**, IL23A, LTB, TLR2, TNFSF13B, VEGFA | 3.1E-3 | 5.6E-1 | 1.9E-1 |

*Total 1686 differentially expressed genes in LGL versus TemRO dataset with FC=2 both up- and down-regulated, p<0.05 (ANOVA), of which 1563 were annotated by DAVID using Affymetrix Human Genome U133 Plus 2.0 array as background and selecting Homo Sapiens as species.

**Adjusted p-value based on Bonferroni and Benjamini-Hochberg correction for multiple testing.

Genes also identified through DAVID LGL versus effector subset and IPA analyses, which are further validated with RQ-PCR are indicated in bold.
