# Supplementary material for: Dysregulated signaling, proliferation and apoptosis impact on the pathogenesis of TCRγδ+ T cell large granular lymphocyte leukemia
Source: PLoS One. 2017 Apr 13;12(4):e0175670. doi: 10.1371/journal.pone.0175670 (PMC5391076; doi:10.1371/journal.pone.0175670)
Supplement: S6 Table — *Relative mRNA expression after ABL housekeeping gene correction. **Median ΔΔCt value of TCRγδ+ T-LGL leukemia patients after correction with average ΔCt values from healthy control samples. ***Fold changes according to supervised TCRγδ+ T-LGL leukemia cells vs. healthy TemRA TCRγδ+ T cells comparison. (DOCX) [file pone.0175670.s007.docx]

**S6 Table. Median (interquartile range) mRNA expressions, median ΔΔCt and microarray fold changes of genes used for RQ-PCR validation.**

| **Gene** | **Healthy controls*** | **TCRγδ+ T-LGL leukemia patients*** | **Median LGL ΔΔCt value**** | **Microarray LGL FC value***** |
| --- | --- | --- | --- | --- |
| ***Validated genes*** | | | | |
| **XIAP** | 3.360 (1.336 – 8.128) | 2.698 (0.5617 – 12.02) | -2.489 | -2.416 |
| **CASP1** | 3.137 (2.056 – 5.116) | 3.060 (1.252 – 6.208) | -5.269 | -2.783 |
| **BCLAF1** | 55.75 (41.62 – 71.92) | 187.7 (10.38 – 600.1) | 3.867 | 2.039 |
| **CFLAR** | 4.452 (4.231 – 4.672) | 0.08167 (0.05796 – 0.2283) | -65.767 | -2.449 |
| **ID3** | 0.006839 (0.001403 – 0.3605) | 2.784 (1.346 – 5.998) | 28.410 | 7.228 |
| **CD28** | 2.700 (1.414 – 5.076) | 1.073 (0.7529 – 5.330) | 3.283 | 7.012 |
| **CCR7** | 1.197 (0.3527 – 3.101) | 0.5301 (0.07773 – 6.065) | 4.372 | 7.377 |
| **CX3CR1** | 52.88 (15.34 – 90.41) | 10.05 (1.092 – 16.23) | -2.044 | -5.433 |
| **IFNG** | 0.5165 (0.3107 – 0.8488) | 0.4938 (0.1770 – 0.8534) | -6.531 | -3.682 |
| ***Non-validated genes*** | | | | |
| **FAS** | 0.5521 (0.3813 – 0.6345) | 1.598 (0.03642 – 2.156) | 3.059 | -2.215 |
| **KLF4** | 2.083 (1.085 – 10.84) | 0.2712 (0.05194 – 0.7014) | -9.363 | 6.813 |
| **LEF1** | 2.764 (0.9663 – 12.62) | 0.4080 (0.2412 – 1.563) | -4.125 | 16.365 |
| **SOX4** | 0.2511 (0.04589 – 0.5471) | 0.1589 (0.03550 – 0.3438) | -32.236 | 13.277 |
| **LTB** | 5.418 (3.768 – 20.24) | 2.839 (0.4984 – 7.019) | -31.265 | 7.388 |
| **PRF1** | 60.35 (33.89 – 84.32) | 125.0 (30.92 – 205.1) | 2.090 | -4.238 |

*Relative mRNA expression after *ABL* housekeeping gene correction.

**Median ΔΔCt value of TCRγδ+ T-LGL leukemia patients after correction with average ΔCt values from healthy control samples.

***Fold changes according to supervised TCRγδ+ T-LGL leukemia cells vs. healthy TemRA TCRγδ+ T cells comparison.
